# Emodin enhances cisplatin-induced cytotoxicity in human bladder cancer cells through ROS elevation and MRP1 downregulation

**DOI:** 10.1186/s12885-016-2640-3

**Published:** 2016-08-02

**Authors:** Xinxing Li, Haolu Wang, Juan Wang, Yuying Chen, Xiaobin Yin, Guiying Shi, Hui Li, Zhiqian Hu, Xiaowen Liang

**Affiliations:** 1 Department of General Surgery, Changzheng Hospital, The Second Military Medical University, 145 S. Fengyang Road, Shanghai, 200003 China; 2 Therapeutics Research Centre, School of Medicine, The University of Queensland, Brisbane, QLD 4012 Australia; 3 Department of Cell Biology, Key Laboratory of the Education Ministry for Cell Differentiation and Apoptosis, Institutes of Medical Sciences, Shanghai Jiao Tong University School of Medicine, 280 S. Chongqing Road, Shanghai, 200025 China; 4 Division of Surgery, Ren Ji Hospital, Shanghai Jiao Tong University School of Medicine, 1630 S. Dongfang Road, Shanghai, 200127 China

**Keywords:** Bladder cancer, Emodin, Cisplatin, ROS, MRP1

## Abstract

**Background:**

Chemoresistance is one of the most leading causes for tumor progression and recurrence of bladder cancer. Reactive oxygen species (ROS) plays a key role in the chemosensitivity of cancer cells. In the present study, emodin (1,3,8-trihydroxy-6-methylanthraquinone) was applied as a ROS generator in combination with cisplatin in T24 and J82 human bladder cancer cells.

**Methods:**

Cell viability and apoptosis rate of different treatment groups were detected by 3-(4,5-dimethylthiazol-2-yl)-2, 5-diphenyltetrazolium bromide (MTT) and flow cytometry (FCM). The expression of transporters was measured at both the transcription and translation levels using PCR and western blotting. In vitro findings were confirmed by in vivo experiments using tumor-bearing mice. The expression of multidrug resistance-associated protein 1 (MRP1) in tumour tissue was measured using immunohistochemistry and side effects of the emodin/cisplatin co-treatment were investigated by histological examination.

**Results:**

Emodin increased the cellular ROS level and effectively enhanced the cisplatin-induced cytotoxicity of T24 and J82 human bladder cancer cells through decreasing glutathione-cisplatin (GSH-cisplatin) conjugates. It blocked the chemoresistance of T24 and J82 cells to cisplatin through suppressing the expression of MRP1. This effect was specific in T24 and J82 cells but not in HCV-29 normal bladder epithelial cells. Consistent with in vitro experiments, emodin/cisplatin co-treatment increased the cell apoptosis and repressed the MRP1 expression in xenograft tumors, and without obvious systemic toxicity.

**Conclusions:**

This study revealed that emodin could increase the cisplatin-induced cytotoxicity against T24 and J82 cells via elevating the cellular ROS level and downregulating MRP1 expression. We suggest that emodin could serve as an effective adjuvant agent for the cisplatin-based chemotherapy of bladder cancer.

**Electronic supplementary material:**

The online version of this article (doi:10.1186/s12885-016-2640-3) contains supplementary material, which is available to authorized users.

## Background

Bladder cancer is the second most commonly diagnosed genitourinary neoplasm, with approximately 357,000 new individuals occurring around the world and about 145,000 people dying from this disease each year [1, 2]. To date, cisplatin-contained chemotherapy is commonly used in patients with advanced or metastatic bladder cancer. Several meta-analysis revealed that cisplatin-based combination chemotherapy could increase the overall survival rate just by 5 ~ 11 % [3, 4]. However, chemoresistance is one of the most leading causes for tumor progression and recurrence of bladder cancer [1]. In non-muscle invasive bladder cancer, 30–80 % of cases will recur and 1–45 % will progress to muscle invasion within 5 years [5]. Thus, it is necessary to reveal the mechanism of chemoresistance and improve the sensitivity of chemotherapy in bladder cancer.

Reactive oxygen species (ROS), such as superoxide free radical hydrogen peroxide or hydroxyl radicals, refer to a series of intermediate products in the process of oxidation-reduction system. The intracellular level of ROS plays a key role in organic metabolism, survival and physiological function [6, 7]. ROS has been found to affect the chemosensitivity of cancer cells [6, 8]. It has been reported that cancer cells can be induced to apoptosis via increasing intracellular ROS generation by anticancer drugs [9]. Zou et al. [10] verified that by increasing intracellular ROS levels, Auranofin induced a lethal endoplasmic reticulum stress response and mitochondrial dysfunction in gastric cancer cells and blockage of ROS production reversed Auranofin-induced endoplasmic reticulum stress, and mitochondrial pathways activation as well as apoptosis. Furthermore, others also reported that increase of ROS generation not only enhanced apoptosis in cancer cell lines, but also exerted the assistant effect in vivo in clinical trials [11, 12]. Our group has found that elevating ROS levels improved the effect of platinum-based chemotherapy drugs against gallbladder cancer [13]. Thus, it is a potential therapeutic strategy to enhance cytotoxicity of drugs by manipulating oxidation-reduction status of cancer cells.

Multidrug resistance proteins are one of the most important factors that cause chemotherapy resistance, which can reduce the therapeutic efficacy and survival for cancer patients [14]. ATP-binding cassette (ABC) family is related to the multiple drug resistance (MDR), which includes P-glycoprotein (P-gp) also named multiple drug resistance 1 (MDR1), multi-resistant related protein family (MRPs) such as MRP1 and MRP2, and breast cancer resistance protein (BCRP) also known as ATP binding cassette subfamily G member 2 (ABCG2). MDR is a serious obstacle in the management of bladder cancer [15]. Therefore, inhibition of multidrug resistance proteins is a potential way to improve the sensitivity of chemotherapy.

Emodin (1,3,8-trihydroxy-6-methylanthraquinone) is a kind of natural anthraquinone contained in the traditional Chinese herbal medicines, especially from the root and rhizome of Rhizoma and Radix families. Emodin plays important roles in anti-inflammatory, antibacterial, diuretic, immunosuppressive and chemopreventive effects [13, 16, 17]. Furthermore, emodin is found to have anticancer effect such as increasing cell apoptosis, cell death and chemotherapeutic sensitization [13]. Emodin can effectively increase levels of ROS and induce apoptosis in many cancer cell lines [13, 16, 18, 19]. We previously reported that emodin potentiated the anticancer effect on gallbladder cancer cells through inhibiting surviving [20]. We further found that besides enhancing apoptosis in cancer cell lines, emodin exerted the adjunctive treatment with chemotherapeutics in vivo [13, 20].

Therefore, based on the above effects of emodin on cancer cells, we hypothesized that emodin could act as an effective agent in bladder cancer. Lin et al. [21] found that emodin induced apoptosis in T24 human bladder cancer cells via the activation of p53, p21, Fas/APO-1, Bax and caspase-3. In this present study, we demonstrated that emodin enhanced cisplatin-induced cytotoxicity through ROS elevation and MRP1 downregulation in T24 and J82 human bladder cancer cells.

## Methods

### Cell culture

The T24 human bladder cancer cells were supplied by the Shanghai Institute of Cell Biology, Chinese Academy of Sciences. The HCV-29 normal bladder epithelial cells and J82 human bladder cancer cells were provided by the Department of Cell Biology, Institutes of Medical Sciences, Shanghai Jiao Tong University School of Medicine. All cell lines were cultured in RPMI 1640 medium (Invitrogen Corp, Carlsbad, CA, USA) supplemented with 10 % fetal bovine serum at 37 °C in a humidified atmosphere containing 5 % CO_2_. Emodin was obtained from Sigma (St. Louis, MO, USA). Glutathione (GSH) assay kit was purchased from Jiancheng Bioengineering Institute (Nan Jing, China). Cisplatin was bought from Qilu Pharmaceutical Co., Ltd. (Nan Jing, China). N-acetylcysteine (NAC), the precursor of GSH, was provided by Sigma (St. Louis, MO, USA). For experiments of 2.3, 2.4, 2.5, 2.6, and 2.7, T24 and HCV-29 cells were treated with emodin (20 μM), cisplatin (1.5 μg/ml), or emodin/cisplatin co-treatment, respectively. J82 cells were treated with emodin (15 μM), cisplatin (1 μg/ml), or emodin/cisplatin co-treatment, respectively.

### Cell viability and apoptosis analysis

Cells were seeded in 96-well plates with 2.0 × 10^4^ cells per well. The cells were incubated with emodin for 24 h at different concentrations (0, 5, 10, 20, 30, 40, 50, 60, 70 μM) and chose the critical concentration (20 μM) treated with cells for 0, 6, 12, 24, 48, 72, 96 h. The cells were incubated with cisplatin for 24 h at different concentrations (0, 0.5, 1.0, 1.5, 2.0, 2.5, 3.0 μg/ml). 3-(4,5-dimethylthiazol-2-yl)-2,5-diphenyltetrazolium bromide (MTT) (Sigma, St. Louis, MO, USA) assay was used to analyze the cell viability as previously described [22]. Cells were treated with drugs for 24 h and apoptotic rates were assessed with flow cytometry using AnnexinV-fluorescein isothiocyanate (AnnexinV-FITC)/propidium iodide (PI) kit (BD Pharmingen, San Diego, CA, USA). Samples were prepared according to the manufacturer’s instruction and analyzed by a flow cytometry (FCM) Calibur (Becton Dickson, San Diego, CA, USA).

### ROS measurement and GSH detection

2,7-Dichlorodihydrofluorescein diacetate (DCFH-DA) method was used for intercellular ROS accumulation [13]. After cells were treated with different regimens, cells were further incubated with 10 mM DCFH-DA for 15mins at 37 °C, with re-incubation of NAC (5 mM) for 4 h, if used. After washed once with ice-cold phosphate buffer saline (PBS), cells were harvested and kept on ice for an immediate detection by FCM. GSH measurement was conducted according to the instruction of assay kit (Jiancheng Bioengineering Institute, Nan Jing, China). The GSH content of the samples was detected as described by Wang et al [19].

### Western blotting

T24 cells were plated in 6 well plates and treated with different regimens for 24 h before lysed in 100 μl of sample solution as previously used by Huang et al [16]. Equal amounts of proteins were electrophoresed on 12 % SDS-polyacrylamide gel and transferred to a nitrocellulose membrane. The membrane was incubated for 1 h in blocking buffer (5 % low-fat milk powder in blocking buffer containing) and then incubated with the mouse antibody against human MDR1, MRP1, MRP2 and ABCG2 (Abcam, Cambridge, UK) at 4 °C for overnight and horseradish peroxidase-conjugated goat anti-mouse immunoglobulin (Sigma, St. Louis, MO, USA) for 1 h before detected by an enhanced chemiluminescence system. The details of antibodies used in this study were shown in Additional file 1: Table S1.

### qPCR and RT-PCR analysis

Total mRNA was extracted from treated cells using trizol reagent (Invitrogen, Carlsbad, CA, USA) according to the instruction of the manufacturer. The cDNA was reverse-transcribed from 2 μg total RNA. β-actin was used as an internal control. For qPCR, detection of PCR products was performed on a Light Cycler system (Roche Applied Science, Basel, Switzerland) using the SYBR Green I kit (TaKaRa Biotechnology, Dalian, China), according to the manufacturer’s instructions. Each sample was done in triplicate. The expression levels of transporters were normalized to β-actin mRNA expression. The RT-PCR was performed as follows: denaturation for 5min at 95 °C, 30cycles of 95 °C for 30s, 55 °C for 45s and 72 °C for 30s, then extended for 10 min at 72 °C. The sequences for β-actin sense and antisense primers were shown in Additional file 2: Table S2.

### MRP1 siRNA transfection

MRP1 siRNA oligonucleotides were transiently transfected, using the Lipofectamine 2000 reagent (Invitrogen, Carlsbad, CA, USA) according to the manufacturer’s instructions with modifications as previously described [23, 24]. In brief, cells were 50 % confluent at the time of transfection. Oligomer-Lipofectamine® 2000 complexes were added to each well containing cells and medium. Mix gently by rocking the plate back and forth. After that, cells were incubated at 37 °C in a CO_2_ incubator for 48 h and medium were changed after 4–6 h. A nonspecific siRNA was transfected as control, which was randomly synthesized and did not correspond to any known gene in the genome database. Forty-eight hours later, cells were lysed for RT-PCR to verify the efficiency of silencing. After that, T24 cells were treated by cisplatin and the rate of cell apoptosis was detected by FCM described above. The siRNA sequences for MRP1 and nonspecific control were shown in Additional file 2: Table S2.

### In vivo study in tumor-bearing mice

All the animal experiments were conducted according to institutional guidelines for animal welfare and animal ethics were approved for all the experiments from the animal committee of the Second Military Medical University. 3 × 10^6^ T24 cells were harvested, washed, and resuspended in serum-free optimum medium and then injected subcutaneously into 6-week old BALB/c-nu/nu mice (*n* = 8 mice per group, purchased from Shanghai Experimental Animal Center, Shanghai, China). Three days after inoculation, the mice were intraperitoneally administered with PBS, emodin (50mg/kg), cisplatin (1mg/kg), or emodin/cisplatin every two days. On day 18, every mouse was sacrificed. After body weight measurement, tumors were isolated, weighted and fixed in 4 % paraformaldehyde (PFA). Hearts, livers and kidneys were stained with Hematoxylin & Eosin to determine the systemic toxicity as described by Li et al [22]. Terminal deoxynucleotidyl transferase(TdT)-mediated dUTP nick end label (TUNEL) assay (Intergen, NY, USA) was performed on paraformaldehyde-fixed and paraffin-embedded tumor sections, using the methods described previously [19].

### Immunohistochemistry

MRP1 expression in tumor tissues was detected via immunohistochemistry. Briefly, all tumors were fixed in 4 % PFA, embedded in paraffin, and then cut into 5-μm paraffin sections for immunohistochemistry. Deparaffinized sections were dehydrated with alcohol series, then incubated with a monoclonal antibody, (Abcam, Cambridge, UK) at 4 °C overnight to detect MRP1 protein. The protein expression was defined as those showing cytomembrane or/and cytoplasm brown staining. Slides were then mounted using an aqueous solution and photographed.

### Statistical analysis

Statistical analyses were performed using SPSS Statistics 17.0 software (SPSS Inc., Chicago, IL, USA) and results were considered statistically significant at *p* < 0.05 (two tailed). Data were shown as mean values ± S.D., and some of the data were displayed in the form of chart. The corresponding experimental figures were drawn using GraphPad Prism v 5.0 software (Graphpad Software Inc, La Jolla, CA, USA).

## Results

### Effects of emodin on T24, J82, and HCV-29 cell viability

**Fig. 1:**
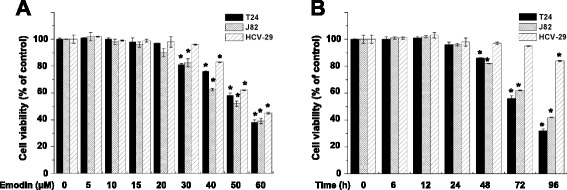
**a** Emodin killed T24 and J82 cells in a dose-dependent manner after 24 h treatment, and was less toxic to HCV-29 cells. **b** Emodin killed T24 and J82 cells in a time-dependent manner, and was less toxic to HCV-29 cells. The emodin concentration is 20 μM for T24 cells, and 15 μM for J82 and HCV-29 cell. Columns, mean of three experiments; bars, S.D. *p < 0.05, experimental group compared with the control group. Each experiment was repeated three times

To investigate the effects of emodin on cell viability, T24 and J82 human bladder cancer cells, and HCV-29 normal bladder epithelial cells were treated with different concentrations of emodin and at different times. Our data showed that emodin killed T24 and J82 cells in the dose-dependent and time-dependent manner, and it was less toxic to HCV-29 cells. The concentration of 20 and 15 μM was selected as appropriate doses for investigating chemotherapeutic sensitivity of T24 and J82 cells at 24 h, respectively (Fig. 1).

### Effects of emodin co-treated with cisplatin on T24 and J82 cells viability

**Fig. 2:**
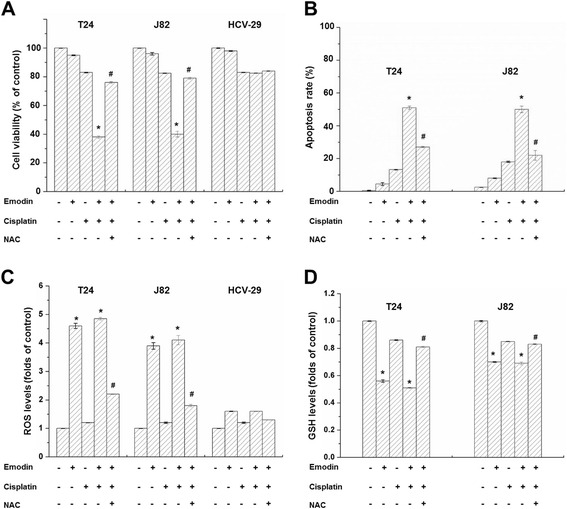
**a** Emodin enhanced the cisplatin-induced cytotoxicity to T24 and J82 human bladder cancer cells, but had little cytotoxicity on HCV-29 normal bladder epithelial cells. The cell viability of T24 cells could be reversed after pre-treatment by NAC for 2 h. **b** Emodin enhanced the cisplatin-induced apoptosis of T24 and J82 human bladder cancer cells, and this effect could be reversed by NAC. **c** Emodin effectively enhanced cellular ROS levels in T24 and J82 human bladder cancer cells, but only slightly elevated ROS levels in HCV-29 normal bladder epithelial cells. **d** Emodin effectively depleted GSH in T24 and J82 cells. The emodin concentration is 20 μM for T24 cells, and 15 μM for J82 and HCV-29 cell. The cisplatin concentration is 1.5 μg/ml for T24 cells, and 1 μg/ml for J82 and HCV-29 cell. Columns, mean of three experiments; bars, S.D. *p < 0.05, experimental group compared with the control group; S.D. #*p* < 0.05, NAC treatment group compared with emodin/cisplatin co-treatment group. Each experiment was repeated three times

According to the cell viability test (Additional file 3: Figure S1 and Additional file 4: Figure S2), the cisplatin concentration of 1.5 μg/ml was selected as an appropriate dose for investigating chemotherapeutic sensitivity at 24 h for T24 cells, and 1 μg/ml for J82 cells. As shown in Fig. 2a, compared with cisplatin or emodin treatment alone, emodin/cisplatin co-treatment could effectively kill T24 and J82 cells. While after pre-treated with NAC, the most commonly used precursor of GSH, for 2 h, the cell viability of T24 and J82 cells could be largely reversed. However, no significant treatment effect was observed on HCV-29 normal bladder epithelial cells. These results suggested that emodin/cisplatin co-treatment selectively killed cancer cells.

### The apoptosis rate of T24 and J82 cells after treatment by cisplatin and emodin

For T24 cells, the apoptotic rate of emodin/cisplatin co-treated group was 51.12 % ± 4.21 %, while that was 10.52 % ± 0.83 % of cisplatin treated group and 3.69 % ± 1.04 % of emodin treated group. In contrast to the cisplatin treated group, the apoptotic rate was 25.14 % ± 1.68 % of NAC treated group (Fig. 2b). The apoptosis of J82 cells after treatment showed the similar trend as T24 cells.

### Detection of cellular ROS and GSH levels

We found that emodin could sharply elevate the cellular ROS levels and depletion of GSH in T24 and J82 cells. While NAC, the precursor of GSH, could effective generate GSH and eliminate ROS (Fig. 2c and d). However, emodin treatment only slightly elevated ROS levels in HCV-29 normal bladder epithelial cells (Fig. 2c), which might be attributed to the complex and effective repair mechanisms of redox system in normal bladder epithelial cells.

### Expressions of transporters

**Fig. 3:**
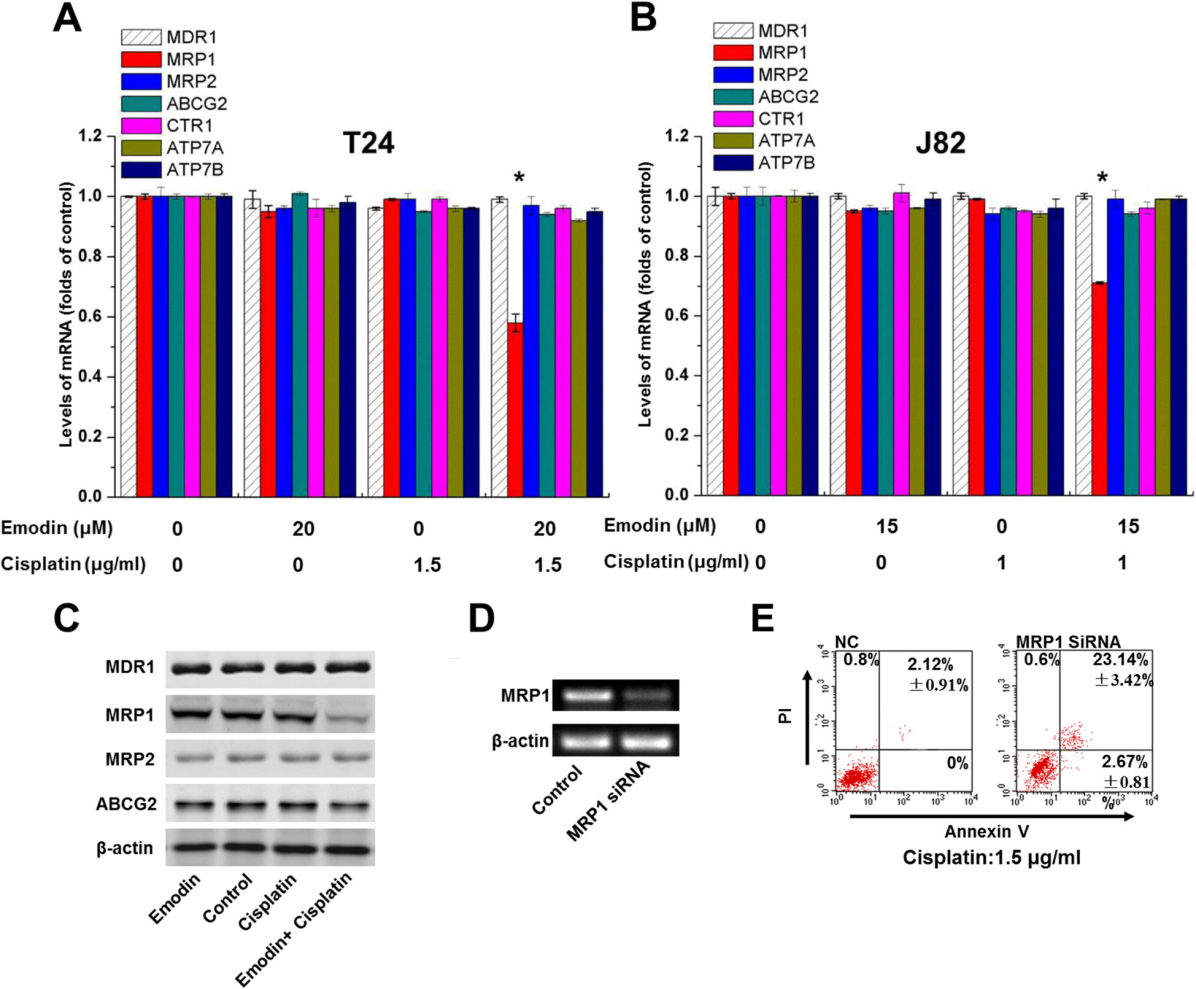
**a** Emodin/cisplatin co-treatment down-regulated the mRNA expression of MRP1, but had no effect on the MDR1, MRP2, ABCG2, CTR1, ATP7A, and ATP7B expression in T24 cells. **b** Emodin/cisplatin co-treatment down-regulated the mRNA expression of MRP1, but had no effect on the MDR1, MRP2, ABCG2, CTR1, ATP7A, and ATP7B expression in J82 cells. **c** Emodin/cisplatin co-treatment inhibited the protein expression of MRP1 but had no effect on the MDR1, MRP2, and ABCG2 expression in T24 cells. After transfected with MRP1 siRNA (**d**), the rate of cisplatin-mediated apoptosis reached 23.14 % ± 3.42 %, which was higher that of the control group (**e**). Each experiment was repeated three times

We measured the mRNA expressions of MDR1, MRP1, MRP2, ABCG2, CTR1, ATP7A, and ATP7B in T24 and J82 bladder cancer cells in different groups. MRP1 expression could be down-regulated at both the transcription level and translation level by emodin/cisplatin co-treatment while MDR1, MRP2, ABCG2, CTR1, ATP7A, and ATP7B did not change in groups (Fig. 3a, b and c).

### The role of MRP1 in cisplatin cytotoxicity

We further verify the role of MRP1 in the sensitivity of bladder cancer cells to cisplatin using MRP1 siRNA transfection. As shown in Fig. 3d and e, after MRP1 silencing, the rate of cisplatin-mediated apoptosis was 23.14 % ± 3.42 %, which was higher than that of the control group (2.12 % ± 0.91 %), suggesting that MRP1 was responsible for the blockade of cisplatin cytotoxicity.

### Effects of emodin co-treated with cisplatin in vivo

**Fig. 4:**
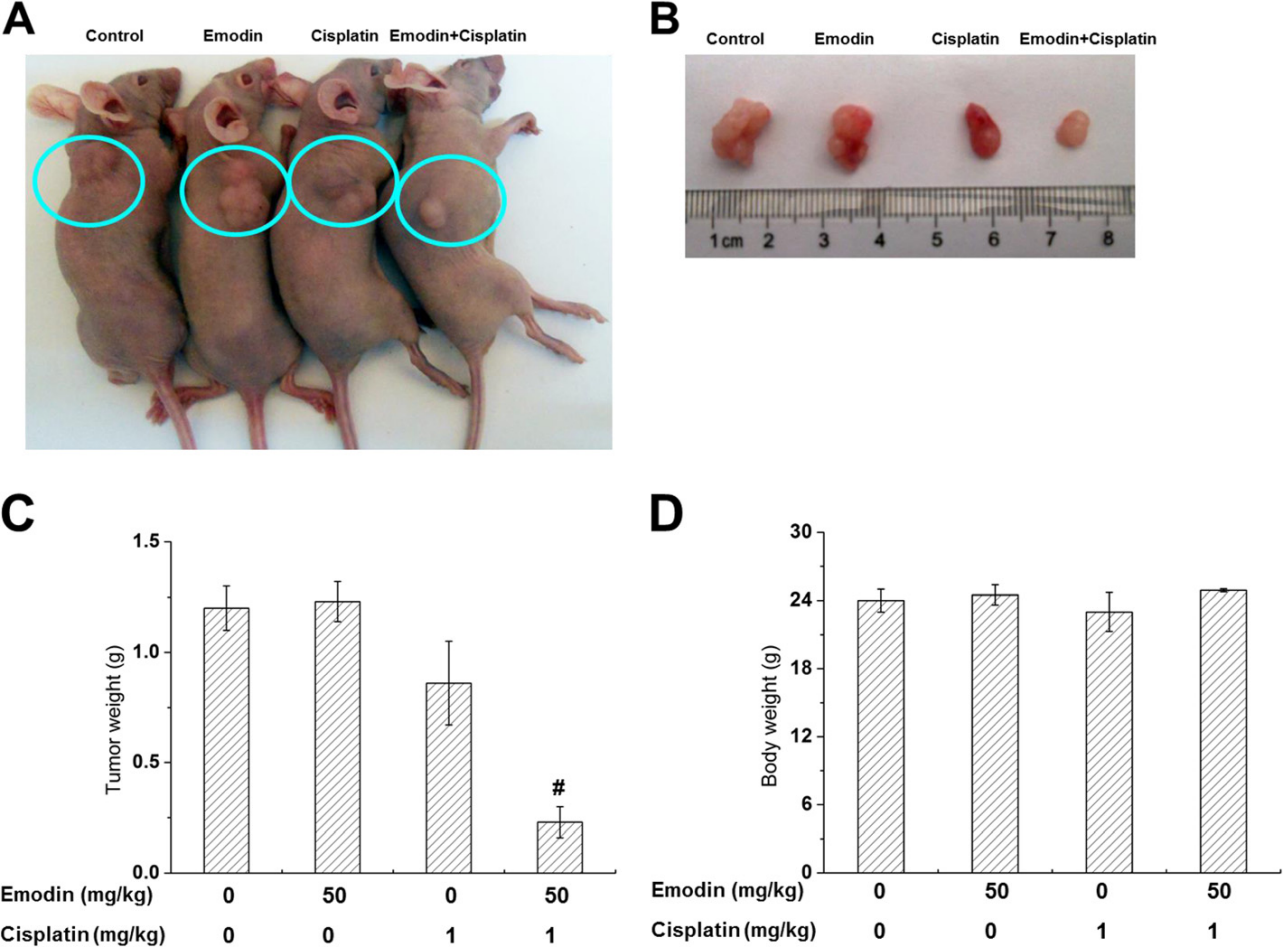
**a** Mice treated with emodin/cisplatin had significantly smaller tumors than those in other group. **b** Transplanted tumors on day 18. **c** Average weight of transplanted tumors. **d** Average body weight of tumor-bearing mice on day 18. Columns, mean; bars, S.D. #*p* < 0.05, emodin/cisplatin co-treatment group compared with cisplatin group (*n* = 8)

As shown in Fig. 4a, b and c, mice treated with emodin and cisplatin had significantly smaller tumors than those from the other groups. In addition, no notable differences on the body weight loss were observed among groups (Fig. 4d) and no obvious necrosis and abnormity were observed in the sections of liver, kidney and heart (Additional file 5: Figure S3). These results demonstrated that emodin/cisplatin co-treatment can significantly suppress tumor growth in vivo with no distinct side effects.

### TUNEL assay in xenograft tumors

**Fig. 5:**
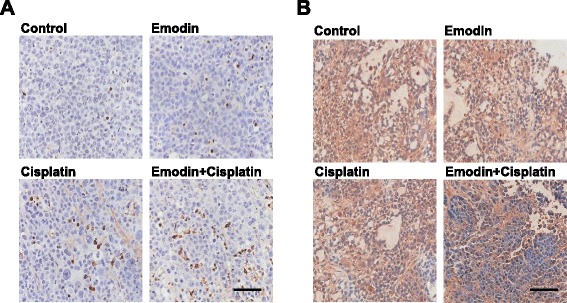
**a** Emodin/cisplatin co-treatment significantly increased cell apoptosis in xenograft tumors. In TUNEL assay of transplantation tissues, the cytoplasmic tan and yellow staining represented positive signal for cell apoptosis, and the nucleus were stained by blue. (*n* = 8). **b** Emodin/cisplatin co-treatment decreased the expression of MRP1 protein in xenograft tumors. In immunohistochemistry of transplantation tissues, the cytoplasmic tan and yellow staining represented positive protein signal for MRP1 protein, and the nucleus were stained by blue. Scale bar: 60 μm (*n* = 8)

Consistent with in vitro experiment, TUNEL assay showed that emodin/cisplatin combination significantly increased cell apoptosis in xenograft tumors (Fig. 5a). Emodin/cisplatin co-treatment group also had lower MRP1 expression than the other groups (Fig. 5b).

## Discussion

Clinical MDR of bladder cancer is caused by a group of integral membrane proteins that transport chemotherapeutics across the cell membrane. [14, 15]. Platinum influx transporters include copper transporter receptor 1 (CTR1) and organic cation transporters. The ABC membrane transport proteins work as drug efflux pumps to decrease the intracellular concentration of anticancer drugs, and it have been reported that increased levels of the drug resistance were associated with adenosine triphosphate ABC transporters. [25–27]. CTR2 and copper-transporting P-type adenosine triphosphates (ATPase’s) have also been founded to affect the MDR of cancer cells [14, 25, 27]. In the present study, we investigated the expressions of MDR1, MRP1, MRP2, CTR1, ATP7A, ATP7B, and ABCG2 in T24 and J82 bladder cancer cells treated with emodin and cisplatin alone or in combination. Our results demonstrated for the first time that MRP1 plays a key role in the chemoresistance of human bladder cancer cells. GSH has been reported to play an important role in MRP1-mediated MDR [28, 29]. Currently, MRP1 is considered as an ATP-dependent GSH conjugate transporter. Different from MDR1, MRP1 phenotype cannot transport platinum drugs without GSH [29, 30]. Its substrates include anionic hydrophilic lipid compounds, especially GSH, glucuronic acid and sulfate conjugations [28–30]. In high MRP1-expressing cancer cells, conjugations of chemotherapeutic drugs combining with GSH can be quickly excreted by MRP1/GSH pump [19]. Cisplatin is one of the most widely used chemotherapeutics [31]. In the present study, we found that T24 and J82 cells were resistant to cisplatin, but emodin can enhance the chemosensitivity of bladder cancer cells to cisplatin by reducing GSH levels or down-regulating the expression of MRP1.

Recently, researchers have found that chemotherapeutic effects often depend on the ROS levels in cancer cells [13, 16, 18, 19]. Cancer cells that are sensitive to chemotherapeutic drugs tend to have higher levels of intracellular ROS, while those with lower levels often exhibit MDR [16]. Thus, the sensitivity of tumor cells to chemotherapeutic agents can be enhanced via selectively elevating levels of ROS in cancer cells [17, 21, 22]. Higher concentration of cisplatin can elevate ROS levels in cancer cells and induce cytotoxicity. However, in the present study, we choose a relatively low concentration of cisplatin (1.5 μg/ml), which could only slightly increase ROS levels without significant cytotoxicity. A low-dose of emodin alone had no significant inhibitory on cell viability to T24 and J82 cells, but could significantly increase the intracellular ROS levels, and enhanced the cisplatin-induced cytotoxicity. Although the emodin/cisplatin co-treatment could also slightly elevate ROS levels in normal bladder epithelial cells, no significant cytotoxicity was observed. This might be attributed to the redox system in normal bladder epithelial cells and effective self-repair mechanisms [32, 33].

**Fig. 6:**
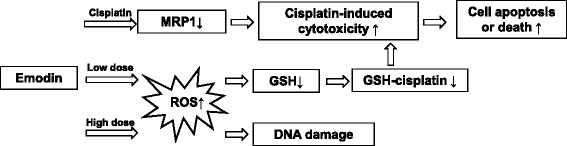
The mechanism of emodin enhanced cisplatin-induced cytotoxicity. Emodin can block the chemoresistance of T24 cells to cisplatin through suppression of MRP1. Low dose of emodin can increase the levels of intracellular ROS and deplete the GSH, resulting in enhanced cisplatin-induced cytotoxicity, while high dose of emodin can directly damage the nuclear DNA of cancer cells

Emodin is a well-studied ROS generating agent and has shown anti-tumor effects including cell cycle arrest, cell proliferation inhibition, apoptosis induction, chemotherapy sensitization, anti-angiogenesis and inhibition of tumor metastasis [17, 21, 34]. Our study showed that emodin can significantly enhance the efficacy of cisplatin-based chemotherapy of bladder cancer both in vitro and in vivo. As shown in Fig. 6, low dose of emodin alone can increase the levels of intracellular ROS and deplete the GSH, resulting in enhanced cisplatin-induced cytotoxicity, while high dose of emodin can directly damage cancer cell DNA. Emodin also blocked the chemoresistance of T24 and J82 cells to cisplatin through MRP1 downregulation. It has been reported that low concentrations of emodin do not affect the viability of cancer cells, but can enhance the pro-apoptosis of arsenic trioxide [18]. Low concentrations of emodin have also been reported to elevate ROS levels in many tumor cells, and enhanced their sensitivity to many chemotherapeutics [16, 19]. Several studies indicated that emodin has anticancer effects in other types of cancer through other mechanisms [35]. In this study, we found that emodin did not have significant systemic toxicity in animals, or cytotoxicity to human normal bladder epithelial cells. Thus, it may be safe and effective as a synergetic chemotherapeutic agent. Although emodin has not been employed in any clinical procedure yet, it has broadened the therapeutic potential to cancer patients, and may be tested in clinic in the near future.

There are some limitations of the present study. First, cancer cell-based xenograft animal models are currently considered with less reliability than orthotopic disease models for the development and design of new therapies. Further preclinical studies are required to confirm our results. Second, although we showed no significant effect of emodin/cisplatin administration on body weight and histological findings of treated mice, it does not indicate emodin has no toxicity. Long-term follow-up are needed to adequately evaluate the toxicity of co-treatment.

## Conclusions

In summary, this study revealed that emodin could increase the cisplatin-induced cytotoxicity against T24 and J82 cells via elevating the cellular ROS level and downregulating MRP1 expression. We suggest that emodin could serve as an effective adjuvant agent for the cisplatin-based chemotherapy of bladder cancer.

## Abbreviations

ABC, ATP-binding cassette; ABCG2, ATP binding cassette subfamily G member 2; AnnexinV-FITC, annexinv-fluorescein isothiocyanate; BCRP, breast cancer resistance protein; DCFH-DA, 2,7-Dichlorodihydrofluorescein diacetate; Emodin, 1,3,8-trihydroxy-6-methylanthraquinone; FCM, flow cytometry; GSH, glutathione; MDR, multiple drug resistance; MRP, multidrug resistance-associated protein; MTT, 3-(4,5-dimethylthiazol-2-yl)-2, 5-diphenyltetrazolium bromide; NAC, N-acetylcysteine; PBS, phosphate buffer saline; P-gp, P-glycoprotein; PI, propidium iodide; ROS, Reactive oxygen species; TUNEL, terminal deoxynucleotidyl transferase(TdT)-mediated dUTP nick end label

## Additional files

Additional file 1: Table S1. Antibodies used in this study. (XLS 7 kb) Additional file 2: Table S2. Primer pairs. (XLS 10 kb) Additional file 3: Figure S1. Cisplatin killed T24 cells in a dose-dependent manner. Columns, mean of three experiments; bars, S.D. **p* < 0.05, experimental group compared with the control group. Each experiment was repeated three times. (PPT 100 kb) Additional file 4: Figure S2. Cisplatin killed J82 cells in a dose-dependent manner. Columns, mean of three experiments; bars, S.D. **p* < 0.05, experimental group compared with the control group. Each experiment was repeated three times. (PPT 94 kb) Additional file 5: Figure S3. No obvious necrosis and abnormity were observed in mouse liver, kidney, and heart by histological examination after H&E stain. Scale bar: 60 μm (*n* = 8). (PPT 1365 kb)
