# Supplementary figures and images for: Emodin enhances cisplatin-induced cytotoxicity in human bladder cancer cells through ROS elevation and MRP1 downregulation
Source: BMC Cancer. 2016 Aug 2;16:578. doi: 10.1186/s12885-016-2640-3 (PMC4971704; doi:10.1186/s12885-016-2640-3)

## Slide 1
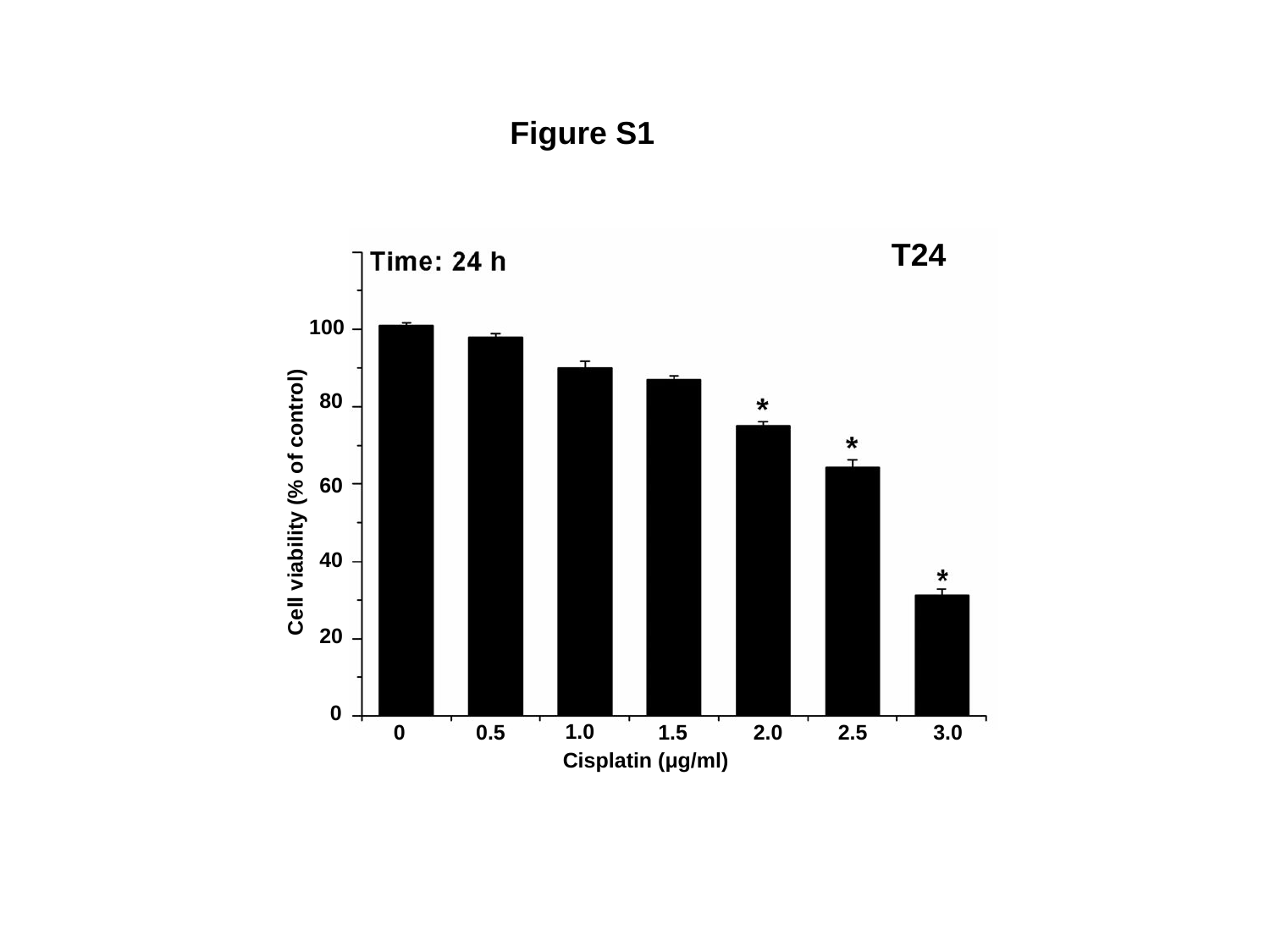

Figure S1
T24
100
80
Cell viability (% of control)
60
40
20
0
1.0
0
0.5
1.5
2.0
2.5
3.0
Cisplatin (μg/ml)

Supplement: Additional file 3: Figure S1. — Cisplatin killed T24 cells in a dose-dependent manner. Columns, mean of three experiments; bars, S.D. *p < 0.05, experimental group compared with the control group. Each experiment was repeated three times. (PPT 100 kb) [file 12885_2016_2640_MOESM3_ESM.ppt]

## Slide 1
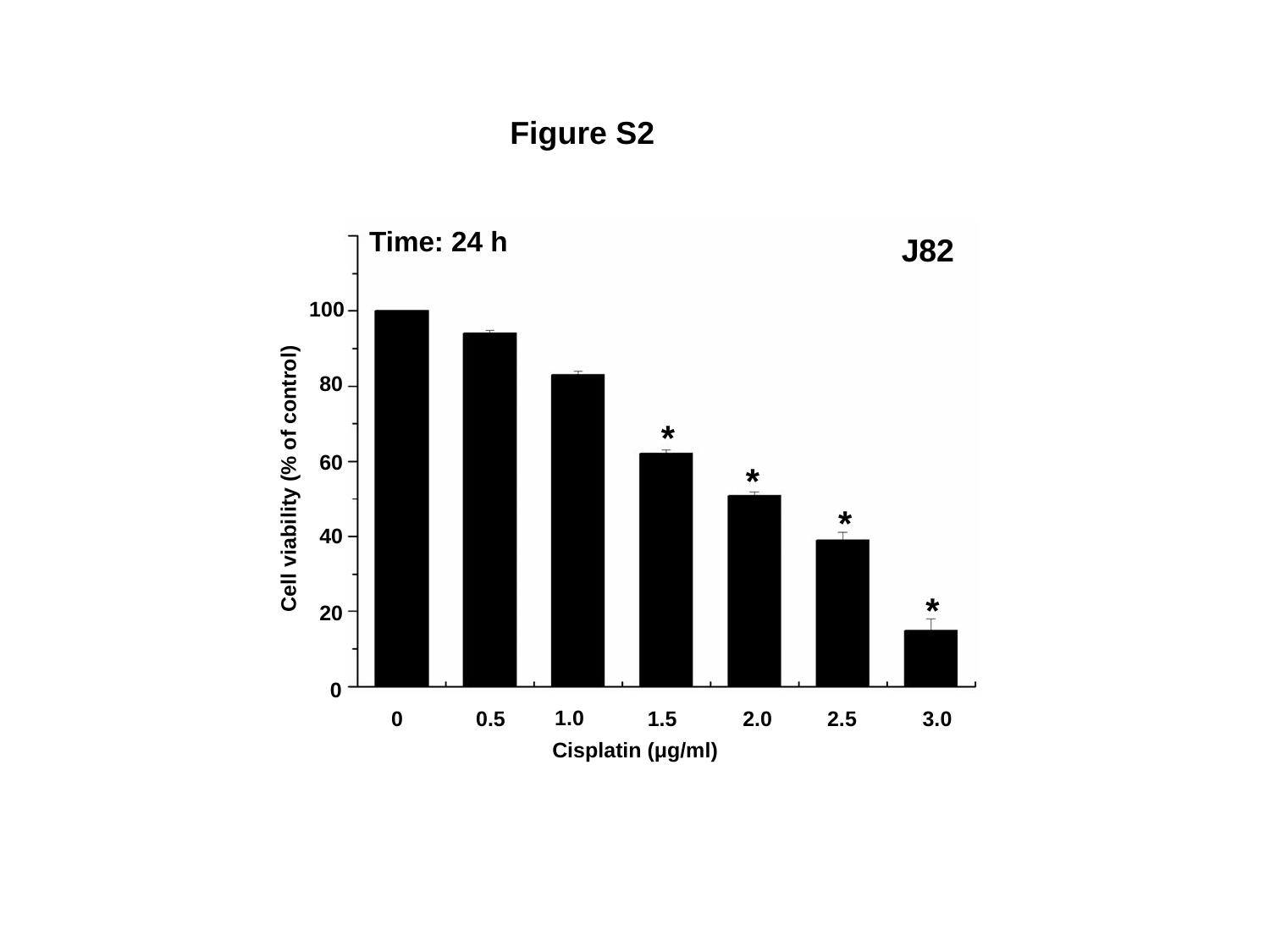

Figure S2
Time: 24 h
J82
100
80
*
Cell viability (% of control)
60
*
*
40
*
20
0
1.0
0
0.5
1.5
2.0
2.5
3.0
Cisplatin (μg/ml)

Supplement: Additional file 4: Figure S2. — Cisplatin killed J82 cells in a dose-dependent manner. Columns, mean of three experiments; bars, S.D. *p < 0.05, experimental group compared with the control group. Each experiment was repeated three times. (PPT 94 kb) [file 12885_2016_2640_MOESM4_ESM.ppt]

## Slide 1
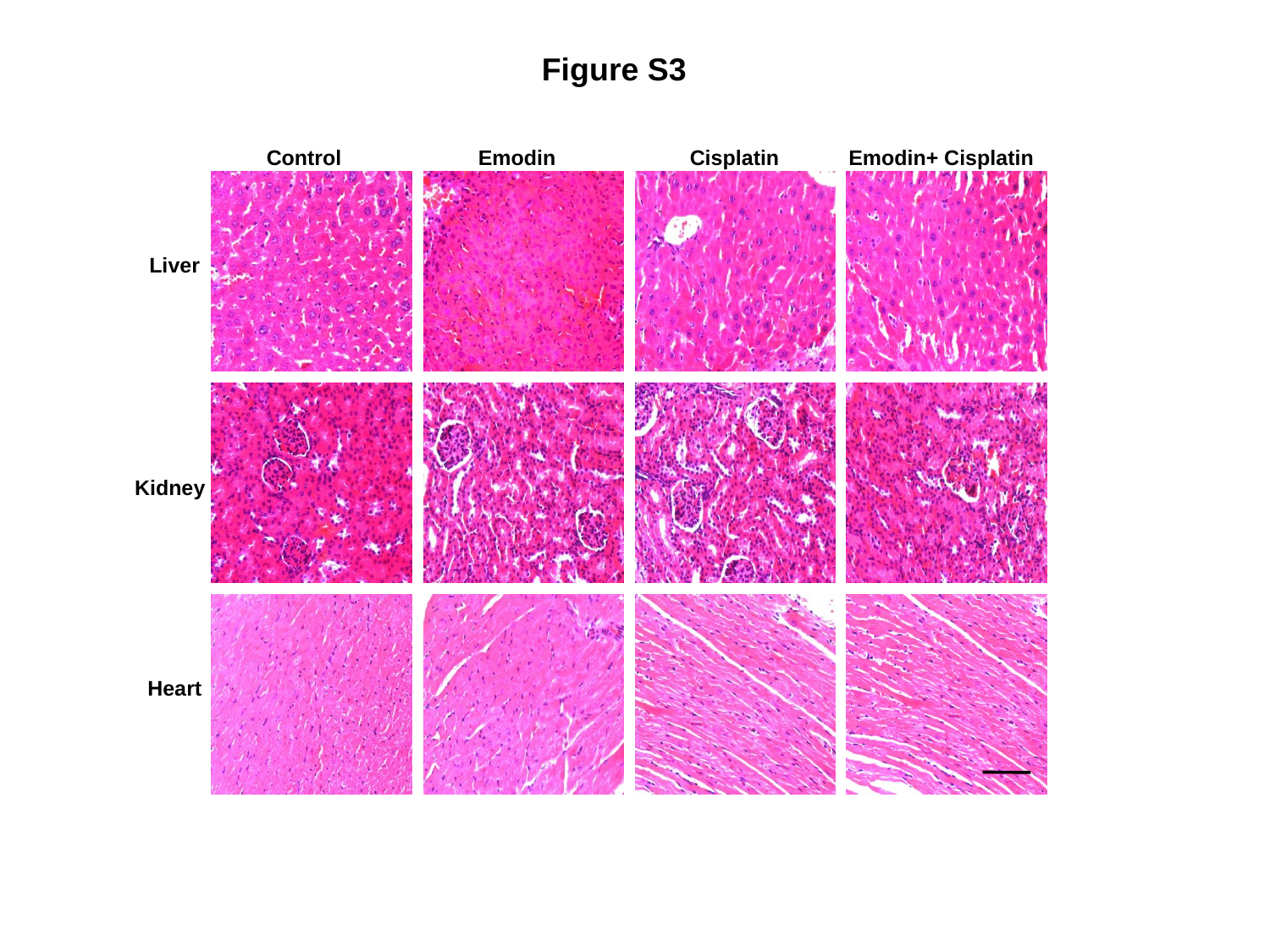

Figure S3
Control
Emodin
Cisplatin
Emodin+ Cisplatin
Liver
Kidney
Heart

Supplement: Additional file 5: Figure S3. — No obvious necrosis and abnormity were observed in mouse liver, kidney, and heart by histological examination after H&E stain. Scale bar: 60 μm (n = 8). (PPT 1365 kb) [file 12885_2016_2640_MOESM5_ESM.ppt]
